# DIFFERENTIAL EFFECT OF LIMITED SOCIAL NETWORK AND SOLITARY LIVING ON HEALTH IMPLICATIONS: PARADOX OF ISOLATION

**DOI:** 10.1093/geroni/igae098.1890

**Published:** 2024-12-31

**Authors:** Hiroshi Murayama

**Affiliations:** Tokyo Metropolitan Institute for Geriatrics and Gerontology, Tokyo Metropolitan Institute for Geriatrics and Gerontology, Tokyo, Japan

## Abstract

In this presentation, I will discuss the distinct effects of a limited social network (SN) and solitary living on health outcomes in later life, drawing on findings from two studies. While both a limited SN and solitary living indicate forms of social isolation, their health implications, especially those associated with solitary living, remain controversial. A limited SN was characterized as engaging in contact with family members living separately or with friends less than once a week, and solitary living was defined as residing without cohabitants. Utilizing extensive cohort data from older adults residing in the Tokyo metropolitan area, we examined the factors modifying the relationship between cognitive decline (CD) and all-cause mortality. The Cox proportional hazard model indicated the CD-mortality association was stronger among those with a small SN than a large SN. Conversely, the association was weaker in living-alone individuals than in cohabiting individuals. The variance in effect was also evident in brain volume metrics. Employing data from a separate cohort study of older Japanese adults, we assessed the impact of social isolation on hippocampal volume changes. Participants with a smaller SN exhibited a more significant reduction in hippocampal volume than those with a larger SN. Surprisingly, solitary living was associated with a lesser decline in hippocampal volume. Although both a small SN and solitary living are indicators of social isolation, they appear to have opposite effects on health. Our results underscore the necessity of differentiating types of isolation when designing and implementing interventions for social isolation.

